# Synthetic Antimicrobial Peptides Exhibit Two Different Binding Mechanisms to the Lipopolysaccharides Isolated from *Pseudomonas aeruginosa* and *Klebsiella pneumoniae*

**DOI:** 10.1155/2014/809283

**Published:** 2014-12-28

**Authors:** Hanbo Chai, William E. Allen, Rickey P. Hicks

**Affiliations:** ^1^Department of Chemistry, East Carolina University, Science and Technology Building, Greenville, NC 27858, USA; ^2^Department of Chemistry and Physics, Georgia Regents University, College of Science and Mathematics, Augusta, GA 30904, USA

## Abstract

Circular dichroism and ^1^H NMR were used to investigate the interactions of a series of synthetic antimicrobial peptides (AMPs) with lipopolysaccharides (LPS) isolated from *Pseudomonas aeruginosa* and *Klebsiella pneumoniae*. Previous CD studies with AMPs containing only three Tic-Oic dipeptide units do not exhibit helical characteristics upon interacting with small unilamellar vesicles (SUVs) consisting of LPS. Increasing the number of Tic-Oic dipeptide units to six resulted in five analogues with CD spectra that exhibited helical characteristics on binding to LPS SUVs. Spectroscopic and in vitro inhibitory data suggest that there are two possible helical conformations resulting from two different AMP-LPS binding mechanisms. Mechanism one involves a helical binding conformation where the AMP binds LPS very strongly and is not efficiently transported across the LPS bilayer resulting in the loss of inhibitory activity. Mechanism two involves a helical binding conformation where the AMP binds LPS very loosely and is efficiently transported across the LPS bilayer resulting in an increase in inhibitory activity. Mechanism three involves a nonhelical binding conformation where the AMP binds LPS very loosely and is efficiently transported across the LPS bilayer resulting in an increase in inhibitory activity.

## 1. Introduction

Because of their novel mechanisms of antibiotic activity, which generally involves some type of membrane disruption, antimicrobial peptides (AMP) have the potential to be developed into useful antibiotic therapeutic agents. Generally AMPs are small highly positively charged [1] amphipathic peptides with well-defined hydrophobic and hydrophilic regions [2–4]. It is generally accepted that the electrostatic interactions that occur between an AMP and the target cell's membrane are the first step in the binding of an AMP to the surface of a cell membrane [5–7]. AMPs exhibit a high net positive charge (+3 to +9) [8] while most bacterial cell membranes contain a relatively high percentage of negatively charged phospholipids as compared to mammalian cells [9]. The resulting difference in the electrostatic nature of the two cell membranes explains, in part, the inherent selectivity of AMPs for bacterial membranes over mammalian membranes [10].

AMPs have evolved in almost every class of living organism, including humans [11], amphibians [12], insects, mammals, birds, fish, and plants [13], as a host defense mechanism against invading microorganisms including bacteria, fungi, protozoa, and parasites [13–15]; they are also considered to be key components in the innate immune response system [16–19]. The antibacterial and anticancer activity of the antimicrobial peptides LL-37 [20, 21], human beta-defensin-3 [21, 22], and other AMPs [21] has been extensively investigated and reviewed in the literature. Particularly beneficial has been the application of solid state NMR methods which have been extensively employed to investigate the interactions that occur between peptides and phospholipid SUVs and LUV phospholipid membrane models [23–29]. Some researchers have suggested that antimicrobial activity is not the primary function of mammalian AMPs such as the defensins [20]. Their primary function may involve immunomodulatory processes in controlling the interaction of acquired and innate immunity [30–33]. The research of Porcelli and coworkers reported the NMR derived structure of the defensin peptide LL-37 bound to DPC micelles [34]. The results obtained were consistent with previous solid state NMR studies that supported a nonpore forming carpet-like mechanism of action for these AMPs [34].

The AMPs developed in our laboratory were designed to be members of the mechanistic class known as membrane-disruptors [19, 35, 36]. In our laboratory we developed a series of AMPs structurally very different from the defensins by incorporating various unnatural amino acids into the primary amino acid sequence with the intent to introduce specific physicochemical properties that will control membrane binding [37]. It is well documented that the selectivity and potency of an AMP against a particular organism are defined in large measure by the complementary nature of the physicochemical surface properties of the AMP and of the target membrane [10, 13, 35, 38–41]. Unnatural amino acids provide a “toolbox” of different physicochemical properties that are not available in peptides composed of the 20 naturally occurring RNA encoded amino acids [42–46]. We have employed this “toolbox” to facilitate the development of peptides with specific physicochemical properties that have the ability to interact with membranes in novel ways [47, 48]. The work of Gottler and coworkers, who previously reported the application of fluorinated analogs of protegrin-1 [49] and other antimicrobial peptides [50] to investigate the role played by changing hydrophobicity on the physical and biological properties of the interactions with lipid membranes and improve activity, can be used as an example of the application of unnatural amino acids to modify biological and physical properties of antimicrobial peptides [49].

Gram-negative bacteria such as* Pseudomonas aeruginosa *[51–56] and* Klebsiella pneumoniae *[57] represent major threats to human health, causing hundreds of thousands of severe infections each year. Infections associated with Gram-negative bacteria are difficult to treat in part because the cell membranes consist of two distinct lipid bilayers of very different chemical compositions [58, 59]. The surface of the outer membrane of Gram-negative bacteria is comprised almost exclusively of negatively charged lipopolysaccharides (LPS) [60–62]. A molecule of LPS is subdivided into three major components, the chemical compositions of the two outer components varying by bacterial strain [63–65]. The outermost component consists of a polysaccharide known as the O-antigen, the core oligosaccharide unit constitutes the middle region, and the innermost portion is the highly conserved phospholipid known as lipid A [63–65]. One of the key functions of LPS is to control the transport of antibiotics, antimicrobial peptides, and host defense proteins into the cell [63, 66–68]. Because of the reduced transport of antimicrobial peptides across the outer membrane, often higher concentrations of the peptide are required to exhibit antibacterial activity against Gram-negative strains than are required to obtain the same level of activity against Gram-positive strains [65]. Therefore, in the case of Gram-negative bacteria, it is critically important to understand the physicochemical interactions that occur between an AMP and LPS in order to design AMPs with increased antibacterial activity against Gram-negative bacteria [58, 60, 64].

The first step in the binding of an AMP to the membranes of Gram-negative bacteria involves the insertion of the AMP into the outer leaflet [69–71] which causes expansion or loosening of the lipid bilayer resulting in the depolarization of the LPS vesicles and allows a transient “self-promoted uptake” pathway to occur, destabilizing the bilayer [72, 73]. This process may be similar to the “carpet-like” mechanism proposed for the binding of AMPs to phospholipid membranes [74–76].

**Figure 1:**
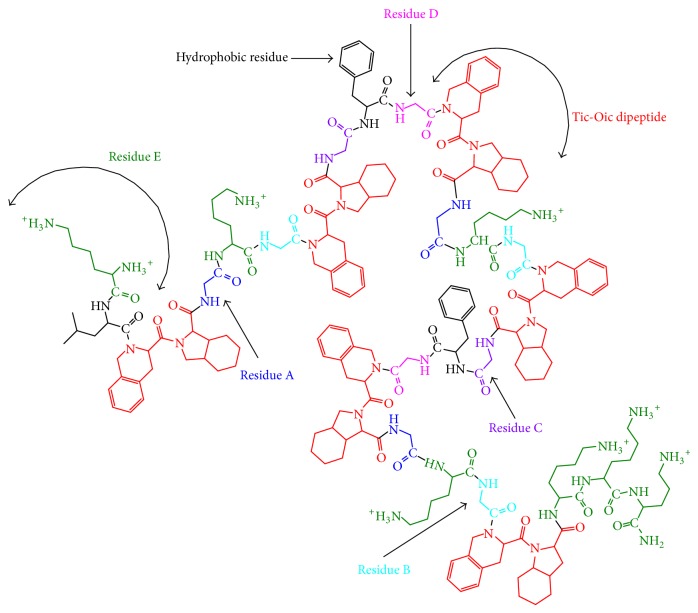
A representation of the residues used in the amino acid sequence of the AMPs under investigation.

The primary amino acid sequence of the AMPs in this investigation incorporates six Tic-Oic dipeptide units, as well as four additional residues (A, B, C, and D) on either side of the intervening hydrophobic and charged residues as shown in Figure 1. These residues define the overall conformational mobility of the peptide backbone. A fifth residue, E, defines the distance between the polypeptide backbone and the positively charged side chain amine group. We have previously shown using electrostatic surface calculations that the distance between the positively charged amino group and the electronegative carbonyl oxygen of the amide bond determines the resulting positive charge density of the side chain [77]. The amino acid residues used for residues A–E are defined in Table 1. The amino acid sequences of the AMPs used in this investigation are listed in Table 2.

We previously reported that increasing the number of Tic-Oic dipeptide units from three to six without the incorporation of residues A, B, C, and D in AMPs 70 or 22 resulted in a dramatic loss in activity against all of the Gram-negative bacteria tested, compared to the analogues containing three Tic-Oic dipeptide units (e.g., AMP 23: Ac-GF-Tic-Oic-GK-Tic-Oic-GF-Tic-Oic-GK-Tic-KKKK-CONH_2_) [78]. (Please see Table 3 for in vitro inhibitory activity of the AMPs investigated in this study).

We propose that the observed differences in inhibitory activity of these AMPs (Table 3) against these two strains of Gram-negative bacteria largely arise from variations in how these peptides interact with the LPS components of the bacteria. To obtain insight into how these AMPs interact with LPS, ^1^H NMR and CD investigations were conducted using SUVs consisting of the LPS isolated from* P. aeruginosa* and* K. pneumonia*.

## 2. Materials and Methods

### 2.1. Peptide Synthesis

Peptide synthesis was performed either manually using t-Boc chemistry or with an automated peptide synthesizer using Fmoc protocols [79, 80] as previously reported [47, 81, 82]. All peptides were purified by reverse phase HPLC [47, 81, 82]. Purified peptides were analyzed again by HPLC and mass spectrometry [47, 82].

### 2.2. Preparation of LPS Liposomes

A 4 mg sample of the appropriate lipopolysaccharide was hydrated with 4 mL of buffer (40 mM sodium phosphate, pH = 6.8) and vortexed extensively. SUVs were prepared by sonication of the milky lipid suspension using a titanium tip ultrasonicator for approximately 10 minutes at a temperature of 40°C until the solution became transparent. The titanium debris was removed by centrifugation at 8,800 ppm for 10 minutes using a table-top centrifuge [6].

### 2.3. Circular Dichroism

Peptide solutions were prepared by dissolving approximately 2 mg of AMPs 70, 74, 75, 79, or 80 in 1.0 mL of phosphate buffer. Due to solubility issues, the CD spectra of AMPs 22, 76, 77, and 78 were collected at a concentration approximately 50% lower than the other AMPs. CD spectra of AMPs 71, 72, and 73 in the presence of either LPS could not be obtained due to precipitation of uncharacterized AMP-LPS complexes. For the LPS liposome studies 350 *μ*L of stock LPS solution was mixed with 50 *μ*L of stock peptide solution. CD spectroscopy is a sensitive technique, so it is commonly used to monitor conformational changes in peptides and proteins [83–85]. However, as LPS can exhibit strong CD absorption, only after careful subtraction of the LPS background signal can meaningful spectra of the AMPs bound to the LPS be obtained [64, 65, 86]. All CD spectra were obtained by acquiring 8 scans using a 0.1 mm cylindrical quartz cell with a spectral range of 260 to 195 nm (at wavelengths below 195 nm the HTV exceeded 400, and therefore data collection was terminated at 195 nm) and a scanning rate of 20 nm/min. Acquisition parameters were bandwidth 1 nm, data pitch 0.2 nm, response time 2.0 s, and 5 mdeg sensitivity. Spectra were collected at room temperature (298 K). Contributions from LPS were eliminated by subtracting from the corresponding AMP-LPS solutions. All analyses of CD spectra were conducted after smoothing with a means-movement function [47, 87, 88]. CD spectra that exhibited HT values of greater than 500 were not used due to excessive light scattering and/or absorption.

### 2.4. NMR

All ^1^H experiments were conducted at 298 K on a Bruker Avance III 400 MHz NMR spectrometer equipped with a 5 mm direct observe Z-gradient broad-band probe. The spectral width was 4,000 Hz, 256 FIDs were collected per experiment. Data were processed using exponential multiplication with a line-broadening function of 5 Hz. Samples contained 1.0 mg/mL of the LPS in the presence of 0.1 mg of the AMP in 600 *μ*L of a 150 mM perdeuterated sodium acetate buffer at a pH of 5.64 in D_2_O.

## 3. Results and Discussion

### 3.1. CD Investigations

**Figure 2:**
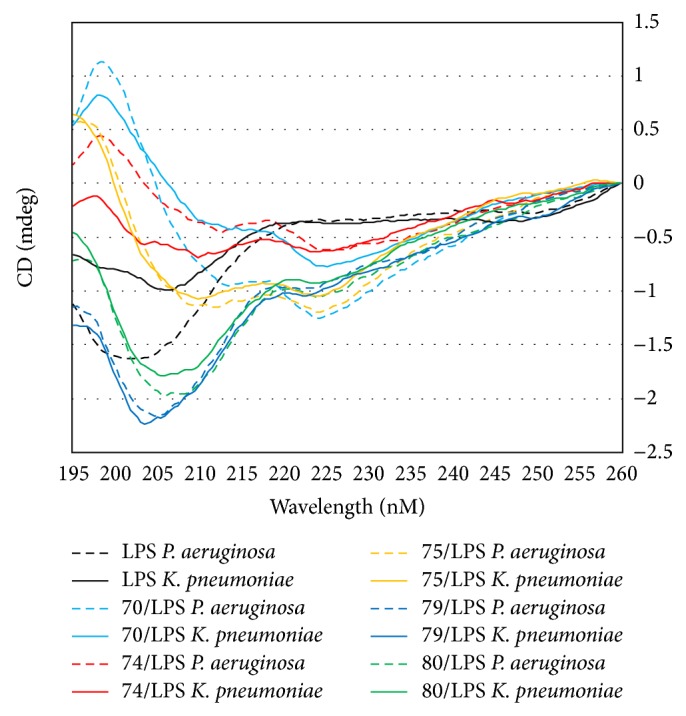
Far-UV circular dichroism spectra of AMPs 70, 74, 75, 79, and 80 in the presence of the LPS isolated from* P. aeruginosa* (dashed lines) and from* K. pneumoniae *(solid lines).

**Figure 3:**
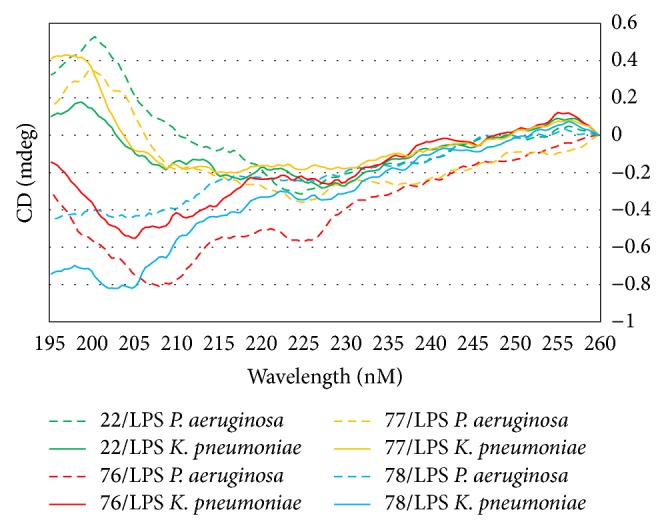
Far-UV circular dichroism spectra of AMPs 22, 76, 77, and 78 in the presence of the LPS isolated from* P. aeruginosa* (dashed lines) and from* K. pneumoniae *(solid lines).

The CD spectra of AMPs 70, 74, 75, 79, and 80 in the presence of LPS isolated from both* P. aeruginosa* and* K. pneumoniae *are shown in Figure 2. The CD spectra of AMPs 22, 76, 77, and 78 in the presence of LPS isolated from both organisms are shown in Figure 3. As can be seen in Figures 2 and 3, the CD spectra fall into two different spectral types. The first exhibited a *λ*_max⁡_ at approximately 198 nm and double *λ*_min⁡_ at approximately 210 and 225 nm. These CD spectra appear similar to those observed for peptides comprised of only the 20 naturally occurring amino acids with predominantly *α*-helical secondary structure. In the case of the peptides under investigation the incorporation of a high percentage of unnatural amino acids means that the traditional methods of characterizing peptide secondary structure by spectral deconvolution are not valid. Therefore, these CD spectra can only be described qualitatively as “helical-like.” In the presence of LPS isolated from* P. aeruginosa*, the CD spectra of AMPs 22, 70, 74, 75, and 77 exhibit helical-like features, while in the presence of LPS isolated from* K. pneumoniae*, the CD spectra of AMPs 22, 70, 75, and 77 (but not 74) exhibit helical-like features. The second type of CD spectra consisting of AMPs 76, 78, 79, and 80 exhibits only negative absorptions with double *λ*_min⁡_ at approximately 204–210 and 225 nm in the presence of the LPS isolated from both bacterial strains. In the presence of LPS isolated from* K. pneumoniae *the CD spectra of AMP 74 also falls into the latter type. The observation of two different types of CD spectra implies that these AMPs adopt two very different sets of conformations on binding to LPS and further suggests two distinct binding mechanisms for these AMPs. The different binding conformations and mechanisms may be explained by the AMPs interacting with different sites or regions of the LPS.

### 3.2. NMR Investigations

Bhunia and coworkers [58] have reported the NMR-derived three dimensional structures of pardaxins Pa1, Pa2, Pa3, and Pa4 bound to LPS micelles. In the pardaxin Pa4-LPS complex, the structure of the peptide was found to be very different from those adopted in the presence of organic solvents and other micelles [58]. These results may provide insight into the structural requirements for selectivity for Gram-negative bacteria, but unfortunately two practical issues prevented us from conducting similar experiments using LPS SUVs with these AMPs. At the concentrations of the AMP required to conduct 2D NMR experiments, the AMP-LPS mixtures precipitated out of solution, and no NMR signals were detected. In addition, the incorporation of six Tic-Oic dipeptide units (which, as secondary amides, lack amide protons) into the sequence of these peptides, coupled with severe overlap of the side chain protons in the region 2.5–1.0 ppm, makes the application of standard homonuclear 2D experiments such as the TOCSY [89, 90] and NOESY [91] very problematic. Consequently, our structural analysis is limited to the use of CD spectroscopy.

**Figure 4:**
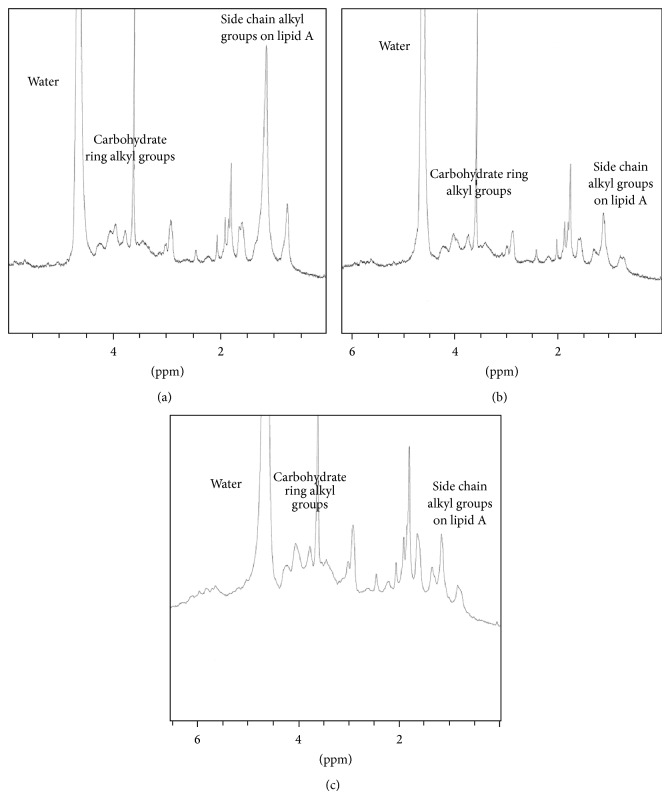
^1^H NMR spectra of (a) LPS isolated from* P. aeruginosa*; (b) AMP 70 in the presence of LPS isolated from* P. aeruginosa*; (c) AMP 79 in the presence of LPS isolated from* P. aeruginosa. *The chemical shift region from 6.0 to 0.0 ppm is shown. Addition of the AMP results in a reduction in peak intensities in the region 2.0 to 0.7 ppm. This region corresponds to the protons on the alkyl side chains of lipid A.

However, one-dimensional ^1^H NMR spectra of AMP-LPS complexes could be employed to monitor changes in the local chemical environments of the LPS as a function of AMP binding. Compared to the ^1^H NMR spectrum of the LPS alone, a significant reduction in the peak heights of the resonances in the region between 1.5 and 0.5 ppm (Figure 4) was observed in the spectra of a 1.0 mg/mL sample of LPS isolated from* P. aeruginosa *as a result of the addition of 0.1 mg of AMPs 70, 74, 75, 79, and 80. (At this low concentration of AMP, no NMR signals corresponding to the AMPs are observed). The region between 1.5 and 0.5 ppm corresponds to the resonances associated with the side chain protons of the lipid A region of LPS. The reduction in peak area indicates a strong binding interaction of these AMPs with this region of lipid A. The region between 4.5 and 3.8 ppm, which corresponds to the polysaccharide resonances of the LPS, exhibits a change in peak position but little change in peak intensity. This indicates a weaker interaction between the AMP and the polysaccharide region of the LPS.

**Figure 5:**
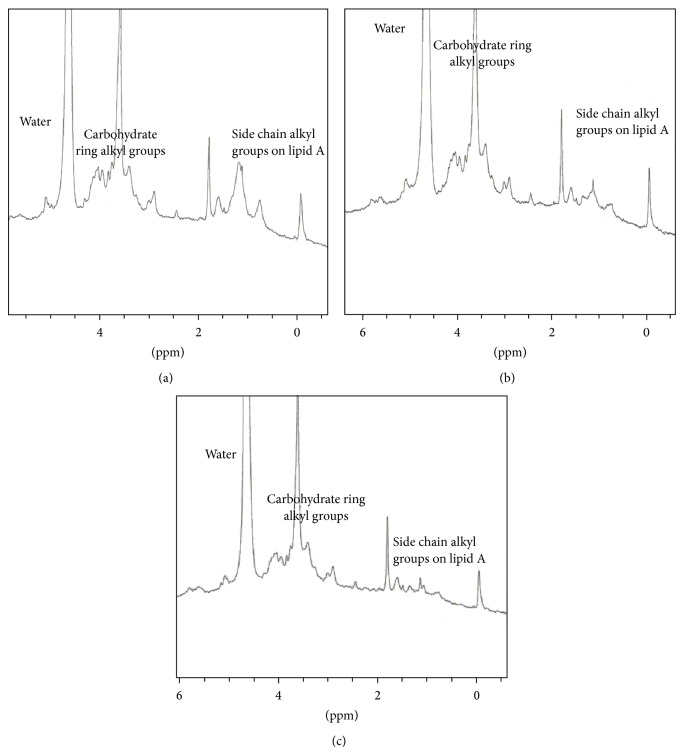
^1^H NMR spectra of (a) LPS isolated from* K. pneumoniae*; (b) AMP 70 in the presence of LPS isolated from* K. pneumoniae*; (c) AMP 79 in the presence of LPS isolated from* K. pneumoniae*. The chemical shift region from 6.0 to 0.0 ppm is shown. Addition of the AMP results in a reduction in peak intensities in the region 2.0 to 0.7 ppm. This region corresponds to the protons on the alkyl side chains of lipid A.

The ^1^H NMR spectra of a 1.0 mg/mL sample of the LPS isolated from* K. pneumoniae *in the presence of 0.1 mg of AMPs 70, 74, 75, 79, and 80 (Figure 5) showed a reduction in the signal intensity as well as changes in the observed chemical shifts in the region of 1.8 to 0.7 ppm relative to LPS alone. The other regions of the NMR spectrum remained unchanged upon addition of these peptides. Such a decrease in peak area would arise from complexation between AMPs and the lipid A region of the LPS isolated from* K. pneumoniae.* These data suggest that the present AMPs exhibit a higher partition coefficient for the lipid A portion than for the polysaccharide or core oligosaccharide of the LPS. This is in accord with the second mechanism of AMP-LPS binding, which involves hydrophobic interactions between the AMP and the hydrocarbon chain region of lipid A [92, 93]. At lower field, between 4.5 and 3.8 ppm, the polysaccharide resonances of the LPS exhibit a change in peak position but little change in peak intensity. This indicates a weaker interaction between the AMP and the polysaccharide region of the LPS.

### 3.3. Proposed Binding Site on LPS

LPS is believed to act as barrier to the transport of material, including drugs, across the outer membrane of Gram-negative bacteria via two mechanisms [72]. The first involves hydrophilic interactions between the substrate to be transported and the densely packed negatively charged oligosaccharide core of LPS [94]. The second mechanism involves sequestering of lipophilic moieties within the hydrocarbon chains of lipid A [92, 93]. The transport of hydrophobic molecules from bulk solvent through the LPS bilayer occurs at a rate that is 98-99% slower than that observed for the transport of the same molecule across a phospholipid bilayer [95, 96]. These two mechanisms indicated that both hydrophobic interactions and electrostatic attractions between an AMP and LPS are possible, this is because AMPs are highly amphipathic, presenting a hydrophobic face and a hydrophilic face to LPS.

Other investigations have been conducted attempting to link the interactions of AMPs with LPS and the observed antibacterial activity against Gram-negative bacteria. For example, the AMP MSI-594 and its mutant analog MSI-594F5A exhibit very different activity against Gram-negative bacteria, with MSI-594 exhibiting greater potency, while exhibiting similar activity against Gram-positive bacteria [65]. Domadia and coworkers reported [65] using NMR that MSI-594 and MSI-594F5A adopt different helical structures in the presence of LPS micelles. MSI-594 adopts a hairpin helical structure, while MSI-594F5A adopts an amphipathic curved helix without the packing interactions that controlled the LPS binding of MSI-594. The differences in the helical conformations adopted by these two AMPs seem to be related to the 3D spatial orientation of the Lys residues [65]. The six Lys residues of MSI-594F5A are on one amphipathic face and are evenly spaced out at a distance of 25 Å [65]. While the six Lys residues of MSI-594 are on one amphipathic face and they are clustered together over a distance of only 17 Å [65]. Domadia and coworkers [65] proposed that, “the compact structure and geometrical compatibility of LPS/MSI-594, provided by the orientation of the side chain of basic residues, could be related to an efficient permeabilization of an LPS membrane of Gram-negative bacteria.” It has been shown that helical content alone does not account for antibacterial activity against Gram-negative bacteria because increasing the helical content of an AMP by incorporation of unnatural *β*-amino acids does not necessarily increase antibacterial activity [97]. As we and others have shown that structure alone is not the defining factor in determining antibacterial activity. It is the three-dimensional character and complementarity of the physicochemical properties, such as charge density and hydrophobicity presented to the cell membranes that define antibacterial activity.

**Figure 6:**
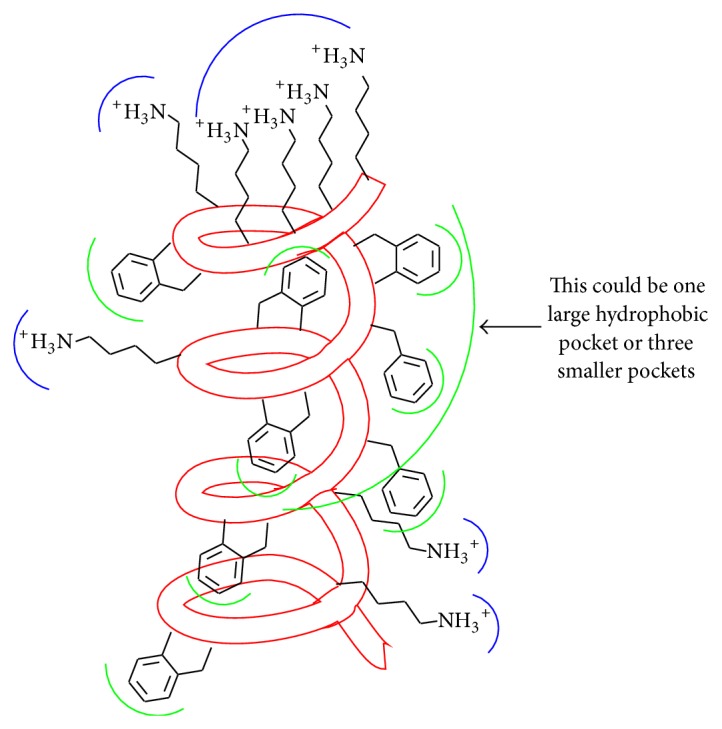
A representation of the proposed AMP-LPS “active site” which is consistent with the AMP adopting a helical conformation upon binding. Blue semicircles represent anionic sites on LPS. Green semicircles represent hydrophobic binding pockets on LPS.

In an effort to explain how the two different spectral shapes observed in the CD spectra for these AMPs relate to inhibitory activity, we propose an LPS-AMP “active site” binding model. The construction of this model is guided by the findings of Domadia and coworkers [65] that the positioning of the Lys residues is critical for transport of the AMP across LPS. We have also incorporated both hydrophobic and electrostatic interactions in our model. A cartoon depiction of a proposed active site that is able to accommodate the helical conformation of the AMP is given in Figure 6. Multiple regions of a single LPS molecule, or multiple LPS molecules, may be required to form the scaffolding of the site. Five cationic residue groupings are present in the AMPs under investigation, and it appears that all five must be paired with negatively charged side chains on LPS for high-affinity binding. This assumption is based on the observation that the CD spectra of analogues containing only three Tic-Oic dipeptide units and three cationic residue groupings, such as AMP 23 (Ac-GF-Tic-Oic-GK-Tic-Oic-GF-Tic-Oic-GK-Tic-KKKK-CONH_2_), do not exhibit helical characteristics in the presence of LPS and exhibit greater in vitro inhibitory activity compared to the larger AMPs under investigation in this study.

Based on the amino acid residues incorporated into these AMPs there may be as many as eight hydrophobic microenvironments (or four localized ones and a single large one) included within the active site on LPS, the number and location of which may vary between bacterial strains. For both strains of bacteria a hydrophobic pocket likely appears at some distance before and after each anionic binding pocket.

This model can be used to explain the observed CD spectra and the inhibitory activity for these AMPs. The CD spectra of AMPs 22 and 70 exhibit helical characteristics in the presence of LPS isolated from both strains of bacteria.* K. pneumoniae, *AMPs 22, and 70 exhibit very poor in vitro inhibitory activity (≥100 *μ*g/mL) against* P. aeruginosa *and* K. pneumoniae*. The combined CD and biological activity data suggests, based on the work of Domadia and coworkers [65], that 22 and 70 adopt helical conformations that bind LPS very strongly and these AMPs are not efficiently transported across the LPS bilayer. AMPs 76, 78, 79, and 80 exhibited CD spectra with nonhelical characteristics as well as an increased in vitro inhibitory activity of 50 *μ*g/mL. The combined CD and biological activity data suggests that 74 adopts a nonhelical conformation that binds LPS very loosely and is efficiently transported across the LPS bilayer. The CD spectra of AMPs 75 and 77 exhibited helical characteristics in the presence of LPS isolated from* P. aeruginosa*; however this is inconsistent with the observed increased in vitro inhibitory activity of 50 *μ*g/mL for these AMPs.

The CD spectra of AMPs 22, 70, and 75 in the presence of LPS isolated from* K. pneumoniae* exhibited helical characteristics of the CD spectra and also exhibited poor in vitro inhibitory activity of ≥100 *μ*g/mL. The combined CD and biological activity data suggests, based on the work of Domadia and coworkers [65], that 22 and 70 adopt helical conformations that bind LPS very strongly and these AMPs are not efficiently transported across the LPS bilayer.

Notably, these two AMPs do not contain any of the four Residues A, B, C, or D. AMP 74 features Gly residues as residue A, increasing the distance between each Lys residue and the following Tic residue. For the LPS isolated from* P. aeruginosa*, the hydrophobic pocket for the active site is probably large enough to accommodate this increase in distance since the CD spectrum of AMP 74 exhibits helical characteristics. The three additional Gly residues will increase the distances between the Lys residues and depending on the conformation adopted by the AMP dramatically alter the three-dimensional spatial orientation of these residues, thus modifying the type of helical structure adopted by 74 on binding to LPS. AMP 74, exhibited very poor in vitro inhibitory activity (≥100 *μ*g/mL) against* P. aeruginosa*. The combined CD and biological activity data suggests, based on the work of Domadia and coworkers [65], that 74 adopts a helical conformation that binds LPS very strongly and this AMP is not efficiently transported across the LPS bilayer. However, the hydrophobic pocket for the active site for the LPS isolated from* K. pneumoniae *appears to be unable to accommodate the increase in distance since the CD spectrum of AMP 74 is not helical in nature. AMP 74 exhibits in vitro inhibitory activity of ≥100 *μ*g/mL against* K. pneumonia. *The combined CD and biological activity data suggests that 74 adopts a nonhelical conformation that binds LPS very loosely and is efficiently transported across the LPS bilayer. AMP 78, contains Gly residues as residue B, resulting in a greater distance between each Lys residue and the preceding Oic residue. The active site hydrophobic pockets for LPS isolated from both* P. aeruginosa *and* K. pneumonia *are incompatible with this increase in distance, as the CD spectra of AMP 78 do not exhibit helical characteristics. AMP 78 exhibits in vitro inhibitory activity of 50 *μ*g/mL against both* P. aeruginosa *and* K. pneumonia. *The combined CD and biological activity data suggests that 78 adopts a nonhelical conformation that binds LPS very loosely and is efficiently transported across the LPS bilayer. AMPs 75 and 77 contain Gly residues as residues C and D; residue C increases the distance from a Phe residue to the following Tic residue and residue D increases the distance from a Phe residue to the preceding Oic residue. Either the hydrophobic pocket for both bacteria is very large and can accommodate the increased molecular bulk of these two modifications, or the proposed pocket plays no role in the binding active site, since the CD spectra of both AMPs in the presence of both LPSs exhibit helical characteristics. The CD spectra of AMPs 75 and 77 exhibited helical characteristics in the presence of LPS isolated from* P. aeruginosa*; however this is inconsistent with the observed increased in vitro inhibitory activity of 50 *μ*g/mL for these AMPs. The two additional Gly residues will increase the distances between the Lys residues and depending on the conformation adopted by the AMP dramatically alter the three-dimensional spatial orientation of these residues, thus modifying the type of helical structures adopted by 75 and 77 on binding to LPS. The combined CD and biological activity data suggests, based on the work of Domadia and coworkers [65], that 75 and 77 adopt helical conformations that bind LPS very strongly and these AMPs are not efficiently transported across the LPS bilayer. The argument for this proposed hydrophobic pocket playing a role in the active site of the LPS isolated from* P. aeruginosa *is provided by AMP 76, which contains Gly residues for residues A and C. Individually these residues are accommodated by the active site binding model, as noted above. However, collectively these residues disfavor binding to the active site as indicated by the nonhelical character of the observed CD spectra. AMP 76 exhibits in vitro inhibitory activity against* P. aeruginosa *of 50 *μ*g/mL. The combined effect of these two residues is to change the relative position of the anionic Lys residues and the hydrophobic Phe residues in three-dimensional space preventing binding to the active site. The data indicate that 76 adopts a nonhelical conformation that binds LPS very loosely and is efficiently transported across the LPS bilayer. AMPs 79 and 80 also appear not to interact with the active site of either LPS since their CD spectra do not exhibit helical characteristics. Both AMPs exhibited in vitro inhibitory activity of 50 *μ*g/mL against both bacteria strains. The data indicate that AMPs 79 and 80 adopt nonhelical conformations that bind LPS very loosely and are efficiently transported across the LPS bilayer.

## 4. Conclusions

This investigation has shown that synthetic AMPs with elongated primary amino acid sequences exhibit helical characteristics in their CD spectra upon binding to SUVs comprised of LPS isolated from either* P. aeruginosa *or* K. pneumoniae. *Data indicate that these AMPs interact with LPS via three different mechanisms. Peptides with CD spectra that exhibit characteristics of helical secondary structure appear to bind to an “active site” on the LPS. In vitro inhibitory data suggest that there are two possible helical conformations resulting from two different AMP-LPS binding mechanisms. Mechanism one involves a helical binding conformation where the AMP binds LPS very strongly and is not efficiently transported across the LPS bilayer resulting in the loss of inhibitory activity. Mechanism two involves a helical binding conformation where the AMP binds LPS very loosely and is efficiently transported across the LPS bilayer resulting in an increase in inhibitory activity. Mechanism three involves a nonhelical binding conformation where the AMP binds LPS very loosely and is efficiently transported across the LPS bilayer resulting in an increase in inhibitory activity.

## Figures and Tables

**Table 1:** Definition of the RESIDUES found in the Six Tic-Oic containing analogs.

AMP number	Residue A^1^	Residue B^2^	Residue C^3^	Residue D^4^	Residue E^5^
22	None	None	None	None	Lys/Arg
70	None	None	None	None	Lys
71	None	None	None	None	Orn
72	None	None	None	None	Dpr
73	None	None	None	None	Dab
74	Gly	None	None	None	Lys
75	None	None	Gly	None	Lys
76	Gly	None	Gly	None	Lys
77	None	None	None	Gly	Lys
78	None	Gly	None	None	Lys
79	None	Gly	None	Gly	Lys
80	None	*β*-Ala	None	Gly	Lys

(1) Residue A is the residue preceding each internal Lys residues (N-terminal side of the Lys).

(2) Residue B is the residue following each internal Lys residues (C-terminal side of the Lys).

(3) Residue C is the residue preceding each internal Phe residue (N-terminal side of the Phe).

(4) Residue D is the residue following each internal Phe residues (C-terminal side of the Phe).

(5) Residue E replaces the charged Lys residues with charged residues with progressively shorter side chains.

**Table 2:** Amino acid sequence of peptide analogs containing six Tic-Oic dipeptide units.

AMP number	Amino acid sequence
22	H_2_N-KL-Tic-Oic-K-Tic-Oic-F-Tic-Oic-K-Tic-Oic-F-Tic-Oic-K-Tic-Oic-KR-CONH_2_
70	Ac-KL-Tic-Oic-K-Tic-Oic-F-Tic-Oic-K-Tic-Oic-F-Tic-Oic-K-Tic-Oic-KKKK-CONH_2_
71	H_2_N-Orn-L-Tic-Oic-Orn-Tic-Oic-F-Tic-Oic-Orn-Tic-Oic-F-Tic-Oic-Orn-Tic-Oic-Orn-Orn-Orn-Orn-CONH_2_
72	H_2_N-Dpr-L-Tic-Oic-Dpr-Tic-Oic-F-Tic-Oic-Dpr-Tic-Oic-F-Tic-Oic-Dpr-Tic-Oic-Dpr-Dpr-Dpr-Dpr-CONH_2_
73	H_2_N-Dab-L-Tic-Oic-Dab-Tic-Oic-F-Tic-Oic-Dab-Tic-Oic-F-Tic-Oic-Dab-Tic-Oic-Dab-Dab-Dab-Dab-CONH_2_
74	H_2_N-KL-Tic-Oic-GK-Tic-Oic-F-Tic-Oic-GK-Tic-Oic-F-Tic-Oic-GK-Tic-Oic-KKKK-CONH_2_
75	H_2_N-KL-Tic-Oic-K-Tic-Oic-GF-Tic-Oic-K-Tic-Oic-GF-Tic-Oic-K-Tic-Oic-KKKK-CONH_2_
76	H_2_N-KL-Tic-Oic-GK-Tic-Oic-GF-Tic-Oic-GK-Tic-Oic-GF-Tic-Oic-GK-Tic-Oic-KKKK-CONH_2_
77	H_2_N-KL-Tic-Oic-K-Tic-Oic-FG-Tic-Oic-K-Tic-Oic-FG-Tic-Oic-K-Tic-Oic-KKKK-CONH_2_
78	H_2_N-KL-Tic-Oic-KG-Tic-Oic-F-Tic-Oic-KG-Tic-Oic-F-Tic-Oic-KG-Tic-Oic-KKKK-CONH_2_
79	H_2_N-KL-Tic-Oic-KG-Tic-Oic-FG-Tic-Oic-KG-Tic-Oic-FG-Tic-Oic-KG-Tic-Oic-KKKK-CONH_2_
80	H_2_N-KL-Tic-Oic-GK-Tic-Oic-βA-F-Tic-Oic-GK-Tic-Oic-βA-F-Tic-Oic-GK-Tic-Oic-KKKK-CONH_2_

**Table 3:** In vitro minimum inhibitory concentration (MIC) and minimum bactericide concentration (MBC) activity against *K. pneumonia *and *P. aeruginosa*.

AMP	*K. pneumoniae* BAMC 07-18		*P. aeruginosa *PAO1	
	MIC (*μ*g/mL)/(*μ*M)	MBC (*μ*g/mL)/(*μ*M)	MIC (*μ*g/mL)/(*μ*M)	MBC (µg/mL)/(*μ*M)
22	>100	>100	>100	>100
70	>100	>100	>100	>100
71	100/*31.2 *	>100	100/*31.2 *	>100
72	100/*33.6 *	100/*33.6 *	50/*16.8 *	100/*33.6 *
73	50/*16.2 *	100/*32.4 *	50/*16.2 *	100/*32.4 *
74	>100	100/*28.6 *	>100	>100
75	>100	100/*29.2 *	50/*14.6 *	50/*14.6 *
76	50/*13.9 *	50/*13.9 *	50/*13.9 *	50/*13.9 *
77	50/*14.6 *	100/*29.2 *	50/*14.6 *	50/*14.6 *
78	50/*14.3 *	100/*28.6 *	50/*14.3 *	50/*14.3 *
79	50/*13.9 *	50/*13.9 *	50/*13.9 *	50/*13.9 *
80	50/*13.8 *	50/*13.8 *	25/*6.9 *	50/*13.8 *

Concentration values for MIC and MBC valves shown in light face are given in *μ*g/mL.

Concentration values for MIC and MBC valves shown in italic font are given in *μ*M.

## Abbreviations

AMP: Antimicrobial peptide
CD: Circular dichroism
Dab: Diaminobutyric acid
DMPC: 1,2-dimyristoyl-sn-glycero-3-phosphocholine
Dpr: Diaminopropionic acid
Fmoc: Fluorenylmethyloxycarbonyl
LPS: Lipopolysaccharides
LUV: Large unilamellar vesicle
MIC: Minimum inhibitory concentration
NMR: Nuclear magnetic resonance
Oic: Octahydroindole carboxylic acid
SDS: Sodium dodecyl sulfate
SUV: Small unilamellar vesicle
t-Boc: Tert-butyloxycarbonyl
Tic: Tetrahydroisoquinoline carboxylic acid.
